# Experiences of community-dwelling older people with dementia participating in a person-centred multidimensional interdisciplinary rehabilitation program

**DOI:** 10.1186/s12877-021-02282-y

**Published:** 2021-06-02

**Authors:** Anna Sondell, Josefine Lampinen, Mia Conradsson, Håkan Littbrand, Undis Englund, Ingeborg Nilsson, Nina Lindelöf

**Affiliations:** 1 Department of Community Medicine and Rehabilitation, Physiotherapy, Umeå University, SE-90187 Umeå, Sweden; 2 Department of Community Medicine and Rehabilitation, Geriatric Medicine, Umeå University, Umeå, Sweden; 3 Department of Community Medicine and Rehabilitation, Occupational Therapy, Umeå University, Umeå, Sweden

**Keywords:** Dementia, Rehabilitation, Interdisciplinary, Experiences, Qualitative research, Aged

## Abstract

**Background:**

There is great need for development of feasible rehabilitation for older people with dementia. Increased understanding of this population’s experiences of rehabilitation participation is therefore important. The aim of this study was to explore the experiences of community-dwelling older people with dementia participating in a person-centred multidimensional interdisciplinary rehabilitation program.

**Methods:**

Sixteen older people with dementia were interviewed about their experiences of participation in a person-centred multidimensional interdisciplinary rehabilitation program. The program comprised assessments by a comprehensive team of rehabilitation professionals followed by a rehabilitation period of 16 weeks, including interventions based on individualized rehabilitation goals conducted with the support of the rehabilitation team. The rehabilitation was performed in the participants’ homes, in the community and at an outpatient clinic, including exercise with social interaction in small groups offered twice a week to all participants. The interviews were conducted at the end of the rehabilitation period and analysed with qualitative content analysis.

**Results:**

The analysis resulted in one overarching theme: *Empowered through participation and togetherness* and four sub-themes: *Being strengthened through challenges*; *Gaining insights, motives, and raising concerns about the future*; *Being seen makes participation worthwhile*; and *Feelings of togetherness in prosperity and adversity*. The participants increased their self-esteem by daring and coping in the rehabilitation. The insights about themselves and their condition motivated them to continue with their prioritized activities, but also raised concerns about how the future would play out. Collaboration in the group and being seen and acknowledged by staff strengthened their own motivation and self-efficacy.

**Conclusion:**

According to community-dwelling older people with dementia, a person-centred multidimensional interdisciplinary rehabilitation program was experienced as viable and beneficial. The participants seemed empowered through the rehabilitation and expressed mostly positive experiences and perceived improvements. Providers of interdisciplinary rehabilitation programs for this group should consider aspects raised by the participants e.g. the positive experience of being challenged in both exercise and daily activities; the importance of being seen and feeling secure; the benefits and challenges of collaboration with others in the same situation; and the generation of new perspectives of current and future situation.

**Supplementary Information:**

The online version contains supplementary material available at 10.1186/s12877-021-02282-y.

## Background

Dementia significantly influences all aspects of life for affected individuals, as well as for family members and friends in their immediate networks [1]. Dementia is the leading cause of dependence in activities of daily living (ADL) among older people [1]. In addition to gradually reduced cognitive function, deterioration of walking and balance abilities is common [2]. The reduced cognitive and functional abilities increase the risks of falls and fractures [3, 4], and the accompanying lack of initiative and interest [5, 6] increases the risk of low levels of social interaction [7] and daily physical activity [8]. Together, these factors can have negative effects on overall health [9, 10]. Other dementia-related consequences that complicate care and have negative effects on health and quality of life include malnutrition, reduced oral health, drug-related problems [11], depression [12], and neuropsychiatric symptoms [1, 13]. The presence of neuropsychiatric symptoms causes a great deal of stress for people with dementia, as well as for informal primary caregivers [14], and is the most common reason why people with dementia move to nursing homes [15]. The symptoms and risks of negative consequences related to dementia implies that this group should be offered individualized rehabilitation during the course of the disease. The achievement of success in interventions may be challenging, especially considering that people with dementia may also have limited awareness of their difficulties in everyday life and anxiety about participation in new situations and contexts [16]. Interdisciplinary team rehabilitation can be a useful approach for managing the complex issues that may be relevant for people with dementia.

Rehabilitation is a set of interventions designed to optimize functioning and reduce disability among individuals with health conditions in interaction with their environment [17]. As rehabilitation seeks to optimize function, activity, and participation, and not to provide a cure, it should be offered to people with dementia despite the neurodegenerative nature of the disease [18]. In contrast to other diseases engaging the central nervous system, e.g. multiple sclerosis [19] and stroke [20], rehabilitation programs are not routinely available for people with dementia in clinical settings [21–23]. Reasons for this situation, in addition to limited resources, may include the challenges involved in managing the complex consequences of dementia, as well as rehabilitation staff’s attitudes regarding the ability of people with dementia to participate in rehabilitation [22, 24]. In addition, people with dementia seem to have less access to rehabilitation in clinical settings after events such as hip fracture or stroke in comparison with people without cognitive impairment [25, 26]. This discrepancy exists despite the findings of several studies demonstrating that the effects of rehabilitation after hip fracture in people with dementia are similar to or greater than those observed in people without cognitive impairment [27–29].

Although studies have evaluated different aspects of rehabilitation for people with dementia, few studies have evaluated rehabilitation provided by interdisciplinary teams comprised of many different professionals who collaborate to conduct assessments, set common goals, and perform continuous follow-ups [30] in an outpatient setting [31–33]. This form of rehabilitation might be a successful approach to managing the complexity of dementia. Thus, interdisciplinary rehabilitation for people with dementia, needs to be further explored. To increase knowledge about feasibility and broaden acceptance of the relevance of interdisciplinary rehabilitation programs for people with dementia, it is important to not only investigate the programs’ impacts, but also to explore the experiences from those participating in such programs. Experiences from participants would also provide important knowledge for future planning and development of rehabilitation programs for older community-dwelling people with dementia. The aim of the present study was to explore the experiences of community-dwelling older people with dementia who took part in a person-centred multidimensional interdisciplinary rehabilitation program.

## Methods

### Study context

This qualitative study was undertaken in the context of the Multidimensional Interdisciplinary Rehabilitation in Dementia (MIDRED) study conducted in Umeå, Sweden in 2016. The MIDRED study is a randomized controlled study evaluating a person-centred multidimensional interdisciplinary rehabilitation program for community-dwelling older people with dementia, including education and counseling of informal primary caregivers. Trial registration: ISRCTN, ISRCTN59155421. http://www.isrctn.com/ISRCTN59155421. (Registered November 3, 2015). Participants in the MIDRED study were recruited through local health centers and the outpatient unit of the Geriatric Center, University Hospital of Umeå. The study inclusion criteria were: diagnosis of dementia according to the 10th revision of the International Statistical Classification of Diseases and Related Health Problems in medical records, age ≥ 60 years, Mini-Mental State Examination (MMSE) score ≥ 10 [34], ability to stand up from a chair with armrests with help from no more than one person, ability to hear and understand Swedish sufficiently to participate in the assessments, expected survival time > 6 months, approval from their physician to participate in the study (physicians were informed about the study), and no initiation of a move to a nursing home. The participants (*n* = 61) were randomized to usual care or to a person-centred multidimensional interdisciplinary rehabilitation program for 16 weeks, including two follow-ups. The present study is a secondary paper from the MIDRED study and the first paper from the trial to be published.

### Participants

Sixteen of the 31 participants in the rehabilitation group were contacted directly or by a phone call by the interviewers and invited to participate in the present interview-based study. The participants were purposefully selected to achieve variation with regard to age, sex, family relationship, physical and cognitive function, depressive symptoms, and also regarding attitudes toward program activities (negative, neutral, positive), and degree of attendance in interventions based on quantitative data (low, medium, high), as estimated by the rehabilitation staff. All 16 participants approached agreed to participate (Table 1). Baseline assessments were made by physiotherapists. The diagnoses were verified by a physician specialized in geriatric medicine according to the Diagnostic and Statistical Manual of Mental Disorders, 4th edition, text revision [38].

**Table 1:** Characteristics of the participants interviewed (*n* = 16)

Age, years	78.5 (63–89)
Women	10 (62.5)
Alzheimer’s disease	9 (56.3)
Mini-Mental State Examination [34], MMSE, (0–30)	22 (15–30)
Berg Balance Scale [35], BBS, (0–56)	51 (33–56)
Functional Independence Measure [36], FIM, (13–91)	78.5 (52–90)
Geriatric Depression Scale 15 [37], GDS-15, (0–15)	2 (0–10)
Living alone	5 (32.3)
Number of years in school	10 (6.5–18)
Adherence, high	12 (75.0)
Attitude, positive	12 (75.0)

Numbers are presented as median (range) or n (%)

For MMSE (cognitive function), BBS (functional balance) and FIM (ADL), a higher score indicates better status. For GDS-15 (depression) a lower score indicates better status. (Instruments not under license)

### Data collection

Three of the authors (NL, IN, and AS) conducted one-to-one semi-structured interviews with open-ended questions in participants’ homes or in a room at the rehabilitations clinic during the last 2 weeks of the rehabilitation. The interviewers were not part of the permanent rehabilitation team staff. To facilitate recall during interviews, the participants were shown pictures of the team staff and the interviewers had brief descriptions of the activities which participants had taken part in and the team staff whom they had met during the rehabilitation. Based on this, the main questions according to an interview guide, were: “What is your experience of taking part in the rehabilitation program?” and “In what way have the activities you participated in affected your everyday life today?”. The participants were encouraged to speak freely in responding to the questions, and the interviews proceeded as conversations. Follow-up questions were asked when necessary. Interview guide developed for this study is provided as Additional file 1.The interviews were audio-recorded, lasted 15–43 min (median 28.5 min), and were transcribed verbatim by a person not involved in the study.

### Data analysis

In this qualitative study, data from individual interviews were analyzed using qualitative content analysis with an inductive approach according to Graneheim and Lundman [39, 40]. This method involves the stepwise, systematic analysis of communication, and a process of interpretation that focuses on similarities and differences that emerge from the material, resulting in the organization of data into categories and themes [39, 40]. This is considered to be an appropriate method for illuminating participants’ lived experiences of a complex phenomenon in a structured manner. The Consolidated Criteria for Reporting Qualitative Research (COREQ) checklist was taken into consideration to ensure transparency [41]. The unit of analysis was all interviews. Two authors (AS, JL) read all the interview transcripts several times to obtain a sense of the whole. They also listened to the audio recordings to obtain further information from interviewees’ tones, voices, and pauses. Next, the transcript content was divided into meaning units consisting of constellations of words and statements with the same meanings. Authors then condensed and coded the meaning units. Based on similarities and differences between codes, preliminary categories were clustered, abstracted, and merged, into ten categories. The interpretive process was mainly conducted by the first and second authors in several steps, with guidance by last and second last author, and yielded one main theme and four sub-themes. At meetings attended by all authors’ creation of categories, sub-themes and theme, were discussed and changes were made until consensus was achieved. The analytical process involved a back-and-forth movement between the whole and parts of the texts.

Trustworthiness was established for example, by all co-authors’ participation in several steps of the analysis, by different pre-understanding between the authors, and by representation of both insider and outsider perspectives. This was discussed in advance in the research group. The disciplines and research areas represented were physiotherapy (AS, NL, HL, and MC), occupational therapy (JL and IN), and physician specialized in geriatric medicine (UE). All authors had experience of working with older people with physical and cognitive impairments, and many (HL, NL, AS, JL, and UE) had experience with person-centred multidimensional interdisciplinary rehabilitation. The authors were involved in the planning of the MIDRED study (HL, MC, NL, IN, and JL), implementation of the rehabilitation (JL and MC, and HL and NL as backup), and performance of the interviews (AS, NL, and IN). Two authors (NL and IN) had extensive experience in interviewing, and in the design and theoretical underpinnings of qualitative content analysis. In this study, we did not apply member check, i.e. when participants check the results for recognition and accuracy.

### The rehabilitation program

The rehabilitation program comprised assessments by a comprehensive team followed by a rehabilitation period of 16 weeks. The team staff included assistant nurse, dental hygienist, dietician, neuropsychologist, nurse, occupational therapist (OT), pharmacist, physician (MD), physiotherapist (PT), and social worker. The team practiced person-centred care, indicating the importance of knowing the person behind the disease, as a human being with reason, will, feelings, resources and needs, in order to engage the person as an active partner in the rehabilitation plan [42]. This team identified problems, needs, and strengths among people with dementia: functional capacity, cognitive function, ADL performance, falls, participation in society, physical activity, nutrition, medical conditions, neuropsychiatric symptoms, and drug use. The findings were used to determine each participant’s individual intervention needs from each professional in the team*.* Representatives of the team staff, together with each participant with dementia and his/her informal caregiver(s), formulated individual rehabilitation goals and planned continuous follow-ups. Based on goals, relevant professionals formed a rehabilitation team for each participant and planned specific interventions. At weekly meetings the professionals evaluated how the rehabilitation were progressing, goal completion, and whether new problems had arisen for the participants. The rehabilitation sessions were conducted at the outpatient facilities at the Geriatric Center (referred to as day rehabilitation unit), in the homes of participants with dementia, and/or in the community. Examples of individually-based interventions were: prescription of cognitive technical devices, support to engage in household activities, introducing participants with dementia to a day-care center and other activities in the community, social support regarding home services and economy, psychological support, review and correction of participants’ medication regimes, support to prevent malnutrition, interventions related to oral health, as well as medical check-ups and controls. The informal primary caregivers were offered six group sessions and individual support when needed.

Each participant with dementia was also offered individualized physical exercise at the day rehabilitation unit twice a week. They travelled to the unit and participated in group exercise sessions followed by a coffee break. An assistant nurse was responsible for organizing transportation to the clinic for all participants, by taxi when needed*.* The assistant nurse and the other staff assisted the participants when arriving and leaving the clinic, and organized the coffee breaks making sure that the participants felt confident and welcomed. The individualized exercise was based on the High-Intensity Functional Exercise (HIFE) Program [43, 44] with the goal of improving muscle strength, balance, and gait ability. The individualized exercise was conducted in a group setting with three or four participants led by two physiotherapists. Participants also received individual recommendations and guidance for the achievement of recommended physical activity levels [45, 46].

## Results

The analysis of the interviews revealed one theme and four sub-themes. The latent message is interpreted in the theme: *Empowered through participation and togetherness*. The sub-themes are as follows: *Being strengthened through challenges*; *Gaining insights, motives, and raising concerns about the future*; *Being seen makes participation worthwhile*; and *Feelings of togetherness in prosperity and adversity* (Table 2). The quotations presented below and related to categories are attributed to participants with fictitious names in parentheses within the text below. Ellipses within the quotes indicate pauses.

**Table 2:** Categories, sub-themes and theme

Category	Sub-theme	Theme
Being challenged is rewarding	Being strengthened through challenges	Empowered through participation and togetherness
Daring and coping provide satisfaction and self-esteem		
Generation of new insights and reflections	Gaining insights, motives, and raising concerns about the future	
Striving to maintain improvements and hoping for continuation		
Fearing the future		
Participation is viewed as a privilege	Being seen makes participation worthwhile	
Sensitivity and support create security		
Perceiving unfulfilled needs and expectations		
Experiencing joy and friendship	Feelings of togetherness in prosperity and adversity	
Perceiving obstacles to interaction		

### Empowered through participation and togetherness

The overall theme reflects that the participants gained empowerment to better handle their situation and everyday life, which was mediated by their experiences of the rehabilitation program. The participants went through a process, prompted by the challenges and expectations during the rehabilitation period in which they progressed from feeling doubt and hesitation to feeling strengthened and competent. Collaboration in the group, and being seen and acknowledged by staff, strengthened their own motivation and self-efficacy.

### Being strengthened through challenges

#### Being challenged is rewarding

Participants initially felt unsure or hesitant about participating in the rehabilitation program, but they gradually changed their minds. They expressed that being challenged was rewarding, when this in turn led to successful management of achievable activities. *“I told her [the OT] that we walked together, me and my husband… she…thought I should try to walk by myself…and it worked...so...if they were afraid that this with my memory...that I would walk astray, but now I have walked so much so all the time when I go out...It’s like a sense of freedom” (Sally).*

There were descriptions of everyday activities that the participants, because of the rehabilitation, felt more capable of doing, or that they could manage again; for example, being able to walk upstairs or rise from a chair without difficulty. They also discovered physical improvements during the exercise sessions as the program progressed. One woman stated that she prevented a fall when vacuuming due to improved balance and leg strength. Participants expressed increased awareness of how much they could push themselves during exercise and in everyday life: *“I can go by myself…I have my hairdresser here and the supermarket...it is not so far away and I manage it” (Sally).*

It was described important that all rehabilitation activities offered were at an appropriate level and adjusted to each individual. They described the physical exercises as individualized and gradually increasing in difficulty to challenge them to improve further. The challenging exercises gave them a positive feeling after exercising: *“It is something very positive when you increase your strength all the time” (*Andrew). Participants were surprised that the exercises made them sweat and gave sore muscles. They realized that they would not have been able to exercise at the same challenging level by themselves since the exercises were sometimes very demanding and felt slightly dangerous.

#### Daring and coping provide satisfaction and self-esteem

Participants perceived themselves as being more competent in daily activities than before the rehabilitation period, which led to feelings of increased self-esteem. One participant mentioned that she had been praised for her progress, and another said that “*I felt I was blossoming again.”* They expressed that they were happier and more satisfied when they dared to do more things, as they did in rehabilitation and in daily life. One woman said*: “When I think about how I would have been if I had not been in the project, I think that I am much more alert and happier and dare to do more things than I would have otherwise*” (Bridget). It was mentioned that they could handle their life situations better and felt joy when others thought they were more capable: *“You maybe become a bit freer...when others think that you are capable”* (Elsa). Another positive aspect that participants mentioned was the opportunity to change their scenery when they travelled to the day rehabilitation unit. They described it as invigorating and reported that going outside their homes by themselves strengthened their self-esteem. *“For me, it is about getting out of the house...because I have been so tied to the house...because I have an illness...and then you become stuck at home…yes...really” (*Nea).

Leaving their partners at home when travelling to the day rehabilitation unit was a positive aspect, as it gave them opportunities to miss their partners: *“I think it is good...it may be good to be apart for a while... I’m there for half a day, so she can find something else to do, then maybe I miss her...ha ha...just a thing like that”* (Eric). It was also expressed that the digital memory aids they received facilitated their everyday lives, for example by reminding them to take their medication or clarifying whether it was day or night: *“It’s perfect...Even if you think you know which day it is, it’s easy to peek and check*” (Boris).

### Gaining insights, motives, and raising concerns about the future

#### Generation of new insights and reflections

The rehabilitation program resulted in new insights and reflections. Participants expressed, for example, that they had the ability and desire to improve their situation, despite their condition: *“You feel that you are good and see that you are at least as good as...the ability to want to become a little better...I am sure it is the same for you”* (Eric). Further, they realized that engaging in regular activities had a positive impact on their mood: *“To feel satisfied when I get home and feel how nice it was that I did this today”* (Karen). It was emphasized that maintaining an exercise routine was easier when they experienced improvements during the rehabilitation process, and that this experience created motivation to continue. Being active and having something to participate in twice a week gave a sense of satisfaction, and was something for them to look forward to. They realized further that they were not alone in having dementia, and that dementia had many manifestations and affected people in different ways. When comparing themselves to others in the group, they reflected on the fact that some were in worse condition than others, which gave them insights that things could be even worse. “*The most important…which you realize afterwards…is that others have problems too, so it’s not just me” (Adam).*

#### Striving to maintain improvements and hoping for continuation

The participants described that they were striving to maintain the improvements that they had achieved during the rehabilitation. It was important for them to take responsibility and to plan to continue exercising after the rehabilitation period, on their own or in another form. Being persistent was described as a positive attribute. One man said*, “I don’t think I have any problem with, so to say, forcing myself to do regular exercise when I notice results so…it’s important that you hang in there” (Boris).*

Some participants had already made plans for continued engagement in activities, and others stated that they were going to continue with previously appreciated activities, such as visiting the library. They were also expected to continue exercising in other regimes, but some felt uncertain about how to carry out activities in the future. Despite help from the staff, others felt unsure about activities in general and about which activities to do, as well as doubts about daring to go to activities in the community by themselves. One woman hoped to include her husband in her training regime for support and togetherness: *“I will try to get my spouse to join me, but he doesn’t want to exercise. He might be able to help me a little... to set things up”* (Bridget).

#### Fearing the future

Participants expressed concerns and uncertainty about the future, which may have been elicited during the course of the rehabilitation. They emphasized that they feared the future and were anxious about what the future might hold for them and how they would end up. Some saw no solution to their situations. Others were afraid about not being able to understand things in the future and about losing the ability to engage in everyday life. They perceived having an incurable and accelerating condition as scary. Furthermore, distressing emotional responses to being in a group of participants in different stages of dementia were expressed. One woman described feeling grief when she compared herself to fellow participants, and when realizing what the next stage of the disease might be: *“I have felt a sadness about being in a group... that is not constructive for me...but constructive for the purpose in this case...I never thought that I would quit or say that I won’t do this, but I already had that feeling that now I’m on the threshold of something that becomes much, much worse”* (Betty).

### Being seen makes participation worthwhile

#### Participation is viewed as a privilege

It was expressed that participation in the rehabilitation was a privilege. Participants stated that they experienced the program as a fantastic and generous venture that gave silver linings to their everyday lives. Participation was described as a great benefit: “*This setup is absolutely fantastic…to be part of it...you get a status of how you are really...it’s incredible to be part of such a group”* (Boris). The participants appreciated the invitation and felt privileged to have been selected for the rehabilitation program. They were also grateful for the ability to contribute to the research project, and felt that it was important to take part in what the rehabilitation program offered. Some experienced no rehabilitation-related change or influence in their everyday lives, but still appreciated participating as they could continue with their previous activities, such as attending lectures or continuing with chi-gong lessons. Others could not express whether any change had occurred, and stated that this was for others to decide.

#### Sensitivity and support create security

The participants appreciated that the staff had appropriate skills, and characteristics, and commended them for their qualities, which contributed to their well-being. The staff’s support and care were sensitive to their individual problems, which created security. The participants emphasized that the rehabilitation team provided great overall service and that the staff had been handpicked for the purpose: *“I think what we have done has been good in many ways…and the people who have been there [the team staff] you feel that they are used to dementia, so you relax…When we sat and talked, it felt like things were normal”* (Bridget). According to the participants, the staff were able to see each person’s progress, and had the ability to adapt treatment to personal needs. *“Yes, they are nice people [the team staff]...they have been attentive...if you ask, you get answers to everything you ask about...helpful in every way…I think that is a suitable team size anyway when doing something like this”* (Adam). The participants appreciated being greeted by staff at the entrance of the rehabilitation unit, which made them feel safe and welcomed. The assistant nurse support with practical things, including coffee breaks, was very much appreciated. Participants described the support with travel as a privilege that solved the logistical problem of getting to and from the day rehabilitation unit, and thus reduced potential feelings of anxiety before leaving home.

#### Perceiving unfulfilled needs and expectations

Some participants stated that they had needs that were not met by the rehabilitation program. For example, wishes for more information about dementia, more help from the doctor, and more strength training were voiced. One woman expressed that she did not feel she was seen as a capable individual, and that the focus of the rehabilitation was on the informal caregivers. There were constructive suggestions for changes of the rehabilitation, such as group allocation based on functional level and stage of the disease. *“We are not alike and have not all progressed as far in our illness, this should be the least you think of there”* (*Eva).* They also had different expectations about the content of the rehabilitation and what it would lead to. One expectation mentioned was to take part in lectures given to informal caregivers instead of being in the exercise session. The different focus between participants and their informal caregivers was also mentioned. One woman had wanted to find a friend during the rehabilitation, but this did not happen. Some described difficulties in adapting to the logistics of traveling between their homes and the day rehabilitation unit and that travel could cause stress and uncertainty.

### Feelings of togetherness in prosperity and adversity

#### Experiencing joy and friendship

The participants described having fun and forming friendships in the group at the day rehabilitation unit. The atmosphere was described as relaxed, supportive, and spirit-boosting. They characterized the group as stimulating, which implied a sense of being comfortable. This comfort, in turn, led them to dare to contribute to the group. *“In the future I think it is important for such activities, that it is a slightly smaller group that stimulates each other, because it is not every day you think positively, there are days that you are negative and you feel down…You play off each other”* (Adam). The coffee break after the exercise was highlighted. Having coffee together and conversing was described as a pleasant experience. Participants felt positive about the conversations during the break, and expressed that everyone felt included and involved in the dialog. They felt able to talk like ordinary people and to discuss current topics. However, when talking about all of these positive aspects, sad feelings emerged that the rehabilitation program would end soon. They expressed that they would miss the activities, the staff and the other participants in the group: *“I will miss the others in the group…I will do that…well I think it has been good…better than when you were alone”* (Bridget).

#### Perceiving obstacles to interaction

The participants also perceived obstacles to interaction in the group setting, and described the group situation as complex given the variation in participants’ abilities and personalities, which meant that it was not always easy to interpret others. The importance of contributing and communicating with each other in the group was emphasized. Some also mentioned feeling sad because they had not gotten to know each other well during the rehabilitation period. Others also described grief about being unable to communicate actively with everyone in the group, or to keep conversations going, as some in the group could not actively engage in the dialogue. *“We sit together around this table, but then...it can be fun if you find a topic of conversation, but it always dies away...it is not possible for these people to respond, but the staff try to keep it going further so we get some views on what we are talking about and so on, but no one comes with their own point of view connected with the topic... if so, it is something entirely different…so the conversation stops from its own lack of…fuel, so to say*” (Betty).

## Discussion

This is to our knowledge the first study exploring experiences of community-dwelling older people with dementia taking part in a person-centred multidimensional interdisciplinary rehabilitation program. The interpretation of the interviews was expressed as the overarching theme *Empowered through participation and togetherness*. Participants described that they were strengthened by facing individualized challenges during the rehabilitation. Mediated by the rehabilitation program, they gained insights about themselves and their condition, which motivated them to continue engagement in prioritized activities, but they also became concerned about how the future would turn out. Participation was experienced as a privilege and being seen made participation worthwhile. They described their experience of being part of the exercise group as togetherness in prosperity and adversity.

Although it was demanding, both cognitively and physically in exercise and daily activities, the participants in our study described the combination of being challenged while supported by the staff as positive. This is in line with positive experiences of being challenged during exercise among people with dementia in nursing homes [47, 48]. Being challenged in exercise, i.e., exercising near maximum capacity [45], is recommended and has also been expressed as a mediator of motivation to continue exercising in community-dwelling people with dementia [49]. The importance of the challenges being individualized and achievable was also emphasized among our participants. By coping with challenges, participants perceived increased self-esteem and feelings of competence. Having something expected of you and being able to accomplish it can potentially result in improvement in mood and self-esteem [48]. Finding satisfaction in accomplishing meaningful activities, has been emphasized in studies with people with dementia previously [50, 51]. Meaningful activities are those that one wants to do, has to do, or needs to do during the day [52] and being able to manage these activities without feeling dependent on others can be important [53]. Engagement in activities fills a void, enhances role identity, and helps people with dementia to express themselves positively [54]. These factors may in turn provide control over self-identity, a critical attribute of selfhood that may endure during the course of the disease [55].

Despite the progressive disease, the participants realized that they were able to influence their situation and improve their physical capacity and ability in daily activities. Like in another study, engaging in regular activities affected the mood positively and being part of a rehabilitation group led to feelings of belonging [56]. The concept of not only exercising, but also offering social interaction while having coffee together, seemed to strengthen the sense of belonging among the participants. The positive experience of this concept has been described earlier by people with dementia [57]. Having a sense of belonging**,** a social arena, being included in enjoyable and meaningful activities, and feeling supported have been expressed as important for the ability to cope with dementia [49, 56–58], and may therefore positively influence health and well-being. Meeting people in the same situation and realizing that they were not alone in having dementia, strengthened them in their situation. The importance of peer-support and the capabilities of people with dementia to engage in meaningful interpersonal interaction have been voiced before [47, 48, 56, 59]. Additionally, engagement in group activities can trigger reflection and adaptation [60], and relationships with other group members and leaders seem to facilitate participation [48].

While some of them were hesitant to participate at first, our participants gradually changed their minds and became positive to participation, which is a similar pattern as the increased motivation seen during exercise over time in nursing home residents [61]. People with dementia may need time to feel safe and embrace new activities and contexts [57]. The participants expressed gratitude for being able to participate in and contribute to the rehabilitation and emphasized the importance of being invested in [48]. This may have contributed to increased motivation and the willingness to make the most of the situation.

Participants in our study praised the professionals, whose sensitivity created security and support along with a welcoming atmosphere. This is in accordance with an activity center study, whose participants valued having the staff seeing them and treating them as normal people [56], and as earlier mentioned found it motivating to engage in exercise [49]. Professional characteristics are important for the success of exercise programs [47, 48] and the forming of a therapeutic alliance [62] is important, according to the authors’ clinical experiences. Physiotherapists participating in an exercise study among people with dementia noted the importance of the ability to take a flexible approach, to engage in personalized communication, and to build successful collaboration [63]. The vast majority of professionals in this study all had experience of working with people with dementia, which probably contributed to the participants’ experiences of the program.

Although there were mostly positive experiences about the group setting and fellow participants, some perceived obstacles to interaction in the groups due to their diverse needs. It seems therefore important that the professionals are supportive and apply their expertise to facilitate the communication in the group settings [49]. The perception that some fellow participants were in worse condition was often experienced positively, but could also raise negative feelings and thoughts about their own future. As suggested by some participants, the composition of groups with more consideration of function and ability, for example a group composed of those in the early stage of the disease might address some of the problems related to group settings. However, dividing persons with dementia into homogeneous groups is a challenge given that it is a heterogeneous progressive disease and symptoms can differ greatly between individuals even early in the course of the disease. Our research group have positive experiences of mixing groups of people with dementia in exercise interventions [47]. The inclusion of people with dementia with different dementia symptoms when offering group activities in clinical settings, requires sensitivity to wishes and needs of each participant**.** Based on the interviews, it was concluded that person-centred care was not always accomplished. This further indicates the importance of sensitivity and effort among staff to increase awareness of each human being and their wishes. There is a need to further evaluate the experience of the composition of different groups when offering activities to people with dementia.

The results show that people with dementia may be empowered through rehabilitation. According to the World Health Organization, patient empowerment is “a process through which people gain greater control over decisions and actions affecting their health” [64] building on core values, positive experiences, possibilities and capabilities rather than problems and symptoms. Being empowered may suggest having improved self-efficacy in one’s daily activities. Self-efficacy is the person’s belief in their ability to succeed in a specific task. It is a critical component of motivation and affects task choices, effort, persistence and achievement [65]. The participants’ self-efficacy could have increased by successful management of activities in the rehabilitation program and in daily life, by watching fellow group members successfully perform exercises, and by leaders’ positive feedback and help to handle feelings [65]. Being empowered can also be interpreted according to the self-determination theory (SDT), a theory of motivation applied in research topics such as physical activity and exercise [66, 67]. The rehabilitation program could have met participants’ essential psychological needs for optimal functioning according to the SDT: autonomy, competence, and relatedness. The person-centred approach with individual goals and the opportunity to leave home to participate in exercise might partly have satisfied the need for autonomy. By daring and coping, and successfully managing exercise and activities, the participants may have gained a feeling of competence. The need for relatedness may have been satisfied by being part of a group led by skilled leaders. Altogether, such need fulfillment could have increased participants’ motivation and led to empowerment.

### Methodological considerations

Some limitations of the study needs to be considered. When interviewing people with dementia, their impaired memory and limited awareness of their disability might influence responses. However, efforts were made to facilitate participants’ recall as the interviews were conducted in close conjunction with the rehabilitation visits, together with the use of recall cues. The sample was selected in the sense that all had agreed to participate in the study. However, none of the participants in this qualitative study had MMSE score of less than 15, which diminishes the transferability to people with more severe dementia.

The interviewers were all observant to the participants’ feelings and whishes during the interviews. The depth and richness of the conversations showed that the participants were able to reflect on and describe participation in the rehabilitation program. Considering participants’ experiences is of great importance when evaluating rehabilitation in general and for people with dementia in particular.

## Conclusion

According to community-dwelling older people with dementia, a person-centred multidimensional interdisciplinary rehabilitation program was experienced as viable and beneficial. The participants seemed empowered through the rehabilitation and expressed mostly positive experiences and perceived improvements. Providers of interdisciplinary rehabilitation programs for this group should consider aspects raised by participants e.g. the positive experience of being challenged in both exercise and daily activities; the importance of being seen and feel secure; the benefits and challenges of collaboration with others in the same situation; and the generation of new perspectives on current and future situation. The findings could furthermore have a positive impact on staff attitudes regarding the ability of this group to participate in rehabilitation. However, more research is needed to explore and evaluate interdisciplinary rehabilitation for people with dementia in various settings, explore experiences of rehabilitation among informal primary caregivers, as well as to determine how to adjust rehabilitation to individual courses of the dementia condition.

## Supplementary Information

**Additional file 1.** Interview Guide

## Data Availability

The datasets generated and analysed during this study are not publicly available to protect the participant’s confidentially. However, they are available from corresponding author upon reasonable request.

## Abbreviations

ADL: Activities of daily living
BBS: Berg Balance Scale
COREQ: The Consolidated Criteria for Reporting Qualitative Research
FIM: Functional Independence Measure
GDS-15: 15 item Geriatric Depression scale
HIFE: The High-Intensity Functional Exercise Program
MD: Medical doctor
MIDRED: The Multidimensional Interdisciplinary Rehabilitation in Dementia study
MMSE: Mini-Mental State Examination
OT: Occupational therapist
PT: Physiotherapist
SDT: The self-determination Theory
