# Supplementary material for: Experiences of community-dwelling older people with dementia participating in a person-centred multidimensional interdisciplinary rehabilitation program
Source: BMC Geriatr. 2021 Jun 2;21:341. doi: 10.1186/s12877-021-02282-y (PMC8173830; doi:10.1186/s12877-021-02282-y)
Supplement: Supplementary file 1 — Additional file 1. Interview Guide [file 12877_2021_2282_MOESM1_ESM.docx]

**INTERVIEW GUIDE**

"You have met these people": *Show photos of the team staff*

• Can you please describe how it has been?

*Talk about what NN has done. Sometimes in the day rehabilitation clinic and sometimes at home. Guide the participant based on the information you have about activities. If necessary, take one activity at a time*.

• How do you experience the participation in these activities/this activity?

• How has the activities you been part in affected your life today?

*Ask your own follow-up questions based on what the conversation will be about.*

*Follow-up questions if necessary:*

• Tell me more, please.

- Can you please describe?
- What? How? Why?

• You said earlier…

*Summarize / reformulate / relate to something NN told.*

• Sometimes you meet in a group (training, coffee, conversation). How do percieve that?

• Sometimes someone from the team come to your home (and to your loved ones). How is that?

*Specify if necessary activity or mention one activity at a time.*

• Is there something special you want to emphasize?

• Is there something that has been particularly good?

• Is there something that has been particularly bad?

• Is there anything you would like to change? If so, what?

*End the interview with a summary and:*

• Have I understood this correctly?

• Is there something you want to add?

(The interview guide was in Swedish and is translated only for this publication)
